# The Role of Structure MRI in Diagnosing Autism

**DOI:** 10.3390/diagnostics12010165

**Published:** 2022-01-11

**Authors:** Mohamed T. Ali, Yaser ElNakieb, Ahmed Elnakib, Ahmed Shalaby, Ali Mahmoud, Mohammed Ghazal, Jawad Yousaf, Hadil Abu Khalifeh, Manuel Casanova, Gregory Barnes, Ayman El-Baz

**Affiliations:** 1 Bioengineering Department, University of Louisville, Louisville, KY 40208, USA; mtali003@louisville.edu (M.T.A.); y.elnakieb@louisville.edu (Y.E.); Ahmed.Elnakib@louisville.edu (A.E.); ahmed.shalaby@louisville.edu (A.S.); ahmahm01@louisville.edu (A.M.); 2 Department of Electrical and Computer Engineering, Abu Dhabi University, Abu Dhabi 59911, United Arab Emirates; Mohammed.Ghazal@louisville.edu (M.G.); jawad.yousaf@adu.ac.ae (J.Y.); Hadil.AbuKhalifeh@adu.ac.ae (H.A.K.); 3 Department of Biomedical Sciences, School of Medicine Greenville, University of South Carolina, Greenville, SC 29425, USA; Manuel.Casanova@louisville.edu; 4 Department of Neurology, Norton Children’s Autism Center, University of Louisville, Louisville, KY 40208, USA; gregory.barnes@louisville.edu

**Keywords:** autism, structure MRI, machine learning, classification, feature selection, hyper-parameter optimization, CAD

## Abstract

This study proposes a Computer-Aided Diagnostic (CAD) system to diagnose subjects with autism spectrum disorder (ASD). The CAD system identifies morphological anomalies within the brain regions of ASD subjects. Cortical features are scored according to their contribution in diagnosing a subject to be ASD or typically developed (TD) based on a trained machine-learning (ML) model. This approach opens the hope for developing a new CAD system for early personalized diagnosis of ASD. We propose a framework to extract the cerebral cortex from structural MRI as well as identifying the altered areas in the cerebral cortex. This framework consists of the following five main steps: (i) extraction of cerebral cortex from structural MRI; (ii) cortical parcellation to a standard atlas; (iii) identifying ASD associated cortical markers; (iv) adjusting feature values according to sex and age; (v) building tailored neuro-atlases to identify ASD; and (vi) artificial neural networks (NN) are trained to classify ASD. The system is tested on the Autism Brain Imaging Data Exchange (ABIDE I) sites achieving an average balanced accuracy score of 97±2%. This paper demonstrates the ability to develop an objective CAD system using structure MRI and tailored neuro-atlases describing specific developmental patterns of the brain in autism.

## 1. Introduction

Autism spectrum disorder (ASD) is a heterogeneous neurodevelopmental disorder that has a strong genetic basis, and various clinical presentations. ASD is characterized by repetitive behavior, social interaction impairments, and difficulties in verbal and nonverbal communication [1]. The prevalence of ASD has been increasing for the last few years, especially in children, reaching almost one in 58 as reported by the Centers for Disease Control and Prevention in the US [2]. A significant financial, and emotional burden faces ASD individuals and their families, as the incidence of ASD increases. Moreover, ASD increases the pressure on the medical, social, and political life of any nation [3].

Since 1943 when Kanner published the first description of ASD, with its association with mild to severe cognitive and behavioral problems in the absence of marked consistent cerebral dysmorphology, has intrigued the medical and scientific world [4]. Therefore, a fundamental goal of any neurobiological study of autism is a description of brain regions that are of abnormal structure or dysfunctional. Once identified abnormalities are characterized, better strategies for the early diagnosis and treatment of autism may follow. Therefore, there is an urgent need for an in-vivo method to study the brain structure at an early age.

### 1.1. Background

Brain studies in-vivo became possible after the invention of Magnetic-Resonance Imaging (MRI). Since the end of 1980s, when Gaffney et al. [5] and Courchesne et al. [6] published the first study of autism using MRI, hundreds of studies have appeared in the literature. Structural MRI (sMRI) examination is widely used to investigate the brain morphology due to its high contrast sensitivity and spatial resolution and because it entails no exposure to ionizing radiation; the last feature is particularly important for children and adolescents [7].

Studies utilized different MRI modalities in order to capture the effect of ASD on brain from different perspectives i.e., sMRI, fMRI, and/or DTI. sMRI was utilized by studies that cared more about the geometry of the cerebral cortex and morphology of the brain [8,9,10]. There are two major types of structural imaging studies depending on the features used: (i) geometric features or (ii) volumetric features. Geometric features are defined as 2D-surface features related to the brain cortex, such as the surface area, circumference, curvature, and thickness [11]. Volumetric features usually refer to the volume of the subcortical structures, such as the hippocampus, putamen, thalamus, etc. [12].

On the other hand, functional MRI (fMRI) was utilized by the studies that investigate alterations in brain activation between ASD and typically developed (TD) groups [13]. As with sMRI, functional imaging studies fall into two broad types: (i) task-based fMRI and (ii) resting-state fMRI. Task-based fMRI is defined as the study of the functional activities and cognitive behaviors of the brain based on the induced stimulus by tasks [14]. In contrast, resting-state fMRI is defined as the study of the functional activities and cognitive behaviors of the brain while the subject is at rest but not sleeping [14].

The third MRI submodality used in ASD studies, and the most recent, is diffusion-tensor imaging (DTI). DTI focuses on the analysis of the structural connectivity of the brain white matter (WM) [15]. DTI characterizes three-dimensional (3D) diffusion of water molecules in biological tissues; it examines normative white matter (WM) development, neurodevelopmental disorders, and neurodegenerative disorders [16,17].

The earliest theories about autism were structurally based. Maybe the most famous, and the earliest, of all theories is the big brain [18,19,20]. The big brain theory assumes that autistic subjects might have a bigger brain volume than their TD peers. Another famous theory about autism, which is rooted in neuropathology but has implications for large-scale anatomy, is the minicolumnar pathology in autism [21].

Furthermore, a recent review article on the post-mortem studies by Fetit et al. found that there are consistent reduction in minicolumn numbers and aberrant myelination of brain tissue in those with ASD [22]. For the readers who are interested in the association between theory of mind and symptoms of ASD, the following article is recommended [23]. Consequently, in this study, we decided to focus on the sMRI submodality.

Most of the studies that were published after Gaffney et al. [5] and Courchesne et al. [6], focused on specific structures in the brain, such as the cerebellum [24,25,26], the amygdala [27,28,29], the hippocampus [27,30,31], and the corpus callosum [32,33,34,35]. Moreover, sMRI provides several means by which researchers can delineate structural changes in the brains of individuals with ASD. Examples of the analytical methods used by researchers to examine brain differences between TD brains and autistic brains are voxel-based morphometry (VBM) and surface-based morphometry (SBM) [8].

VBM-based studies are defined as the studies that have two principal features: tissue density and tissue volume [9]. SBM studies focus on the intrinsic topology of the cerebral cortex. The intrinsic topology of the cerebral cortex is considered as 2-D sheet with a highly folded and curved geometry; that topology cannot be measured directly with VBM. Therefore, SBM provides information complementary to VBM [8].

The proposed study is an SBM study. Thus, we will be focusing on the morphological features of the brain. Evidence supports that several aspects of cerebral morphology are different between autistic brains and TD brains [1,10,36,37]. Moreover, the issue with examples of the cerebral morphology that differ between ASD brains and TD brain would be: (i) the cortical thickness as it reflects dendritic arboziation [38], (ii) the cortical surface area, which is linked to the number of minicolumns in the cortical layer [39], (iii) the cortical folding pattern, which may reflect an abnormal pattern of intrinsic as well as extrinsic connectivity [40].

Therefore, multifactorial etiologies of ASD can be examined by studying the relationship among such multiple cortical features [41]. Multiple SBM studies were performed using the morphology of the brain to find statistical differences between TD and ASD brains [42,43,44,45]. Other studies focused on building ASD predictive models using different machine learning (ML) algorithms [41,46,47,48]. In the following section, we are going to briefly review some of those articles along with their results.

### 1.2. Related Work

#### 1.2.1. Statistical Studies

Recent evidence suggests that the developmental trajectory of the cortex in ASD is significantly different from those of TD individuals. Among statistical analysis studies, Levitt et al. [10] proposed a 3-dimensional (3D) mapping of cortical sulcal patterns in autism. The authors recruited 21 ASD and 20 TD subjects, all within the age of (7–13) years old. They reported statistically significant difference in the anterior and superior shifting of the superior frontal sulci bilaterally, anterior shifting of the right sylvian fissure, the superior temporal sulcus, and the left inferior frontal sulcus in the autistic group relative to the normal group.

The authors suggested that those findings indicate delayed maturation in autistic subjects’ brain regions, which are involved in diverse functions, including memory, emotion processing, language, and eye gaze, which is consistent with delayed myelination patterns seen on MRI in ASD. Nordahl et al. [36] tested the abnormalities in cortical shapes using a SBM across a range of ASD with age between 7.5 years old to 18 years old. The authors subdivided the ASD subjects into three groups: low-function ASD, high functioning ASD, and Asperger’s syndrome. The authors reported that the low-functioning ASD group had a prominent shape abnormality centered on the pars opercularis of the inferior frontal gyrus.

The high-functioning ASD group had bilateral shape abnormalities similar to the low-functioning group but smaller in size and centered more posteriorly, in and near the parietal operculum and ventral postcentral gyrus. The Asperger’s syndrome subjects had bilateral abnormalities in the intraparietal sulcus. Moreover, they reported that all the abnormalities located in the cortex are identified across all ages; however, they were more pronounced in the children. The authors concluded that these findings are consistent with the evidence of an altered trajectory or multiple trajectories of early brain development in autism, and this study identified several regions that may have abnormal patterns of connectivity as a result of the altered trajectories in individuals with autism.

Studies of cortical thickness support the hypothesis that altered trajectory or trajectories of early brain development are localized in particular regions within the ASD brain. Nunes et al. [37] proposed a longitudinal study utilizing Autism Brain Imaging Data Exchange (ABIDE) I and ABIDE II data sets. The authors explored the age-related changes in cortical thickness in TD and ASD population within age range 6–30 years old. They reported that there are no overall group differences in cortical thickness (CT) across the entire age ranges; however, the ASD and TD populations differed in terms of age-related changes.

Those changes were located primarily in the frontal and temporal-parietal areas. They concluded that the most reliable feature for localizing atypically developed brain areas in ASD is the linear slope of CT (curvature). Khundrakpam et al. [1] proposed a study to solve the inconsistent evidence of cortical abnormalities in ASD. They utilized the ABIDE I dataset as it solves the problem of small sample size. After applying quality control and a specific inclusion criterion, the authors included 560 subjects out of 1100 available subjects.

The authors computed age-specific differences in the cortical thickness in ASD and the relationship of any such differences to the symptom severity of ASD. The authors reported an increased cortical thickness in ASD, primarily left lateralized, from six years onwards, with differences diminishing during adulthood. These data highlight the dynamic nature of morphological abnormalities in ASD and highlight the importance of studies across brain development (ages) to understand the altered regional trajectories compared to TD individuals. The readers who seek additional information about the neural circuits associated with ASD are advised to read the review article by Benjamin E Yerys and John D Herrington [49].

#### 1.2.2. Machine Learning/Predictive Studies

In addition to statistical analysis studies, other studies attempted to classify ASD using classical ML models. Dekhil et al. [46] utilized features from both sMRI and fMRI modalities. Morphological features included from the sMRI module were the (i) surface area, (ii) volume, (iii) thickness, (iv) curvature, and (v) folding index. For the fMRI module, the features included were the Person correlation coefficients between time courses of different brain regions. For both modalities, the Desikan–Killiany (DK) atlas was utilized to parcellate brain regions. The authors utilized 185 subjects obtained from the National Database for Autism Research (NDAR).

The authors reported 75% classification accuracy using fMRI data only, 79% classification accuracy using sMRI data only, and 81% when fusing both features together. Elnakieb et al. [50] proposed a similar framework to diagnose ASD using DTI imaging. Elnakieb et al. successfully achieved a maximum accuracy of 99% with five-fold cross-validation while working on five ABIDE II dataset sites that provide DTI imaging data. Yassin et al.  [48] proposed different ML to perform multi-class classification among TD, ASD, and schizophrenia subjects, and a binary classification between each pair of classes.

They had a sample size of 36 ASD, 106 TD, and 64 schizophrenia subjects. They extracted CT, subcortical structure volumes, and surface area. The classification was performed by support vector machine (SVM), logistic regression (LR), random forest (RF), decision tree (DT), K-nearest neighbors (KNN), and adaptive boosting (AB) classifiers. The authors reported 69% accuracy for the multi-class classification, 75% for ASD vs. schizophrenia classification, 75.8% for ASD vs. TD classification, and 70.6% for schizophrenia vs. TD classification. The authors concluded that SVM, and LR were the best performing classifiers. Cortical thickness and subcortical volume-based classification had better performance across different diagnostic labels and classifiers when compared with the surface area.

Ecker [41] proposed a ML model utilizing a set of five morphological features including both volumetric features (concavity, curvature, fold index) and geometric features (surface area, CT) at each spatial location on the cortical surface. A total of 20 TD and 20 ASD subject were recruited for the study. The authors reported accuracy of 85% using SVM. For additional information regarding utilizing ML with MRI modalities to diagnose ASD and other disorders, such as Alzheimer’s, the reader is advised to read the following articles [47,51].

### 1.3. Summary of the Aims and Limitations of the Work

In this study, we propose a comprehensive ML model to detect imaging markers for autism and then utilize these imaging markers to train a set of linear and non-linear classifiers to distinguish between ASD and TD. The main motivations behind using solely morphological features that are extracted from the brain cortex while neglecting the subcortical structures are (i) the segmentation of subcortical structures is more challenging and prone to error more than the cortex segmentation, and (ii) most of the significant findings in the literature are achieved by utilizing the SBM methods. The proposed model defines a global neuro-atlas annotating all the brain regions associated with ASD among all subjects of the data set, as well as a local neuro-atlas for each site independently.

Neuro-atlases are built via employing sophisticated a machine-learning algorithm utilizing different classifiers, recursive feature elimination with cross-validation (RFECV) using four different classifiers (SVM with linear kernel (LSVM), RF, LR with $l_{1}$-norm (Lg1), and LR with $l_{2}$-norm (Lg2)). Eventually, a training step via three linear and five non-linear classifiers. Furthermore, the proposed model utilizes features extracted only from the brain cortex, such as the curvature, CT, surface area, and volume. In this study, we attempted to avoid the limitations existed in the aforementioned literature, such as working on a small sample size [41,48], or neglecting the heterogeneity of the ASD [13,46].

Furthermore, as it was previously mentioned that age is a significant confounding variable on ASD, we adjusted our data to account for the effect of age on the extracted brain features. Consequently, in the proposed study, we are utilizing the ABIDE I data set [52], which comprises 1112 subjects collected from different sites/hospitals in the US. In this work, we are answering the question of whether a site-based classification, i.e., local model, would improve the classification accuracy over a one ML model for the whole set. Moreover, we study if there would be any common features between the selected features from each site, and the selected feature from the global model.

In order to answer the aforementioned questions, two implementations were carried out for the proposed model: (i) on each site of the ABIDE I dataset separately to find local the local neuro-atlas of each site, and (ii) on the whole dataset to find the global global neuro-atlas for the whole autism spectrum represented by the available subjects. The main contribution of the proposed work can be summarized as follows: (i) building a comprehensive ML pipeline to find morphological features and brain regions that are correlated with autism, (ii) finding the anomalous neuro-circuits caused by autism (e.g., neuro-atlases), and (iii) investigating a global ML model that can be used to diagnose ASD subjects with different demographics and scanning parameters.

## 2. Materials and Methods

A comprehensive ML pipeline is proposed in this study to select morphological features and brain regions that relates to ASD. The ML pipeline starts with downloading the sMRI volumes of ASD and TD subjects provided by ABIDE I dataset [53], then the preprocessing of the sMRI volumes is performed by Freesurfer V.6.0 [54,55,56,57]. Preprocessing consists of three stages, which are: (i) intensity normalization, (ii) skull stripping, and (iii) brain segmentation. Each of the aforementioned stages comprises a set of substages, which are going to be briefly discussed in the following sections. After preprocessing, features are extracted in the form of two numerical representation for each morphological feature for each brain region. A data matrix, and a target vector are created and passed to feature selection algorithm to select the candidate imaging markers. Reduced data matrix based on the candidate imaging markers, and the target vector are then passed to the ML algorithms to select the best ML model that can be used for classifying ASD and TD subjects.

The whole pipeline is automated with Python 3.7 [58]. We utilized pandas as the data manipulation package [59], numpy and scipy for numerical analysis and matrices operations [60], scipy for performing statistical tests [61], nibabel for reading and writing Freesurfer files [62]

**Figure 1:**
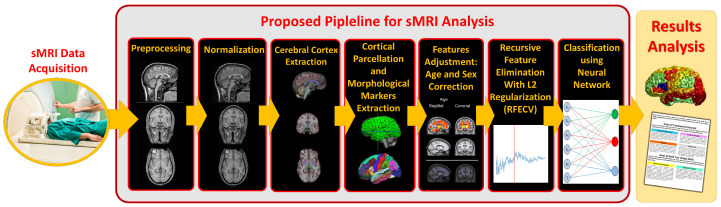
Overview of the proposed system starting from acquiring MRI volumes up to the diagnosis.

Figure 1 demonstrates the general block diagram of the proposed model for each of the global model, and the local model. For the global model, the proposed block diagram is applied only one time over the whole dataset. On the other hand, for the local model, the proposed block diagram is applied on each independent site. Results of both the global model, and each site of the local models are analyzed and compared to each other. Each site’s results using the local model answers the research question about findings of local imaging markers. Similarly, the global model answers the research question about finding global imaging markers for the all the subjects included in the dataset. In the following sections, each of the main blocks in both models is discussed in detail.

### 2.1. Dataset

ABIDE I is a famous publicly available dataset. Using ABIDE I achieves two-fold advantages: (i) It facilitates replicating the results, since it is publicly available. (ii) It comprises a large sample size, which adds more significance to the findings. ABIDE I contains sMRI and resting-state fMRI data acquired on individuals with ASD and TD individuals from 17 independent sites (please see the Supplementary Material S1). ABIDE I includes 1112 subjects divided into 530 subjects with ASD, and 573 subjects with TD.

The original studies included in ABIDE received approval from each participating site’s Institutional Review Board (IRB). All sites diagnosed autism using the Autism Diagnostic Interview-Revised (ADI-R), or Autism Diagnostic Observation Schedule (ADOS). Moreover, each site provided basic phenotypic data on each subject, including age, sex, and intelligence Quotient (IQ). For more details about ABIDE, refer to [52].

### 2.2. Pre-Processing

Preprocessing is a crucial requirement to eliminate the between-subjects variability that may stem from data acquisition, different scanners, artifacts, or partial volume effects. Moreover, brain MRI scans usually contain non-brain tissues as it is shown in Figure 1. FreeSurfer performs multiple steps on each sMRI volume to extract the morphological features. Those steps are intensity normalization, brain extraction and skull stripping, brain segmentation and area labeling, tessellation of the gray-white matter boundary, surface inflation and spherical atlas registration, and eventually cortical surface parcellation to the Desikan–Killiany (DK) atlas.

It is worth noting that the main assumption behind the preprocessing is that as long as FreeSurfer succeeds in extracting the morphological features of the cerebral cortex and parcelate them to DK, then confounding variables relevant to the MRI scanner wont be major concern. This assumption is based on the fact that FreeSurfer outputs the morphological features in their physical unit e.g., mm, mm^2^, mm^3^.

#### 2.2.1. Intensity Normalization

Variations in both intensity and contrast across sMRI images, resulting in the corruption of the sMRI images, are typically due to magnetic susceptibility artifacts and RF-field inhomogeneities. This corruption is undesirable for any segmentation procedure, which utilizes intensity information in order to classify voxel data into different tissue types [63]. To correct the aforementioned corruption, the following procedure of 11 steps is repeated with iterating oversteps from (viii)–(x).

The procedures are: (i) Construct a set of histograms from overlapping slices parallel to the x-y Cartesian plane in the magnetic co-ordinate system. (ii) Smooth the resulting histograms using a fairly broad Gaussian window. (iii) Use a peak-finding algorithm to determine the mean white matter intensity. (iv) Discard the outliers from the array of the detected mean white matter intensities. (v) Fit a set of cubic splines to the resulting coefficients of the valid slices. (vi) Use the splines to interpolate the coefficients for each point along the *z* axis. (vii) Adjust each intensity value by the coefficient at its z coordinate. (viii) Find all points in the volume that are at the center of a 5×5×5 neighborhood of intensity values that all lie within 10% of the white matter peak. (ix) Build a Voronoi diagram and set all voxels unassigned in step (viii) to the correction value of the nearest control point. (x) Perform a few iterations of “soap-bubble” smoothing. (xi) Scale the intensity at each voxel in the volume by the computed correction field [64].

The results are shown in Figure 1, the visual transformation of the brightness level between the preprocessing step and the normalization step is to reduce the variance of brightness for the same tissue inter-subjects due to different data acquisition methods. For more mathematical and implementation details, the reader is referred to [64,65].

#### 2.2.2. Brain Extraction or Skull Stripping

Brain extraction of skull stripping is the process of automatically strip the skull (or any non-brain tissue) from the intensity normalized image. In order to remove the skull and any non-brain tissue, a tessellated ellipsoidal template is deformed into the shape of the inner surface in the skull. Two kind of forces drive the deformation process: (i) An MRI-based force, and (ii) A curvature reducing force.

The MRI-based force is designed to drive the template outward from the brain. It is calculated based on nonlocal information obtained by sampling the MRI data along the surface normal to each vertex of the template tessellation. The curvature reducing force enforces a smoothness constraint on the deformed template, which can be seen as encoding *a priori* knowledge about the smoothness of the inner surface of the skull [64]. The result of this step is illustrated in Figure 1.

#### 2.2.3. Brain Segmentation & Area Labeling

The segmentation process is a two-step procedure: (i) A preliminary classification is performed based solely on the intensity information, and (ii) This volume is examined and the regions that contain more than one tissue type are marked for further processing [64]. After segmentation, a 3D surface reconstruction and brain parcellation to an anatomical atlas is performed on the segmented volume. The 3D surface reconstruction is performed via 2 steps: (i) tessellation of the gray-white matter boundary as described in [54,66], and (ii) surface inflation and spherical atlas registration as described in [54,65].

Brain parcellation to an anatomical atlas, which is the Desikan–Killiany (DK) atlas, is described in [57]. DK atlas parcellates the brain into 68 cortical labels, 34 for each hemisphere. The results of the segmentation and the DK atlas parcellation are shown in Figure 1. For more detailed information on each of the aforementioned preprocessing steps, the reader is referred to the following publication [53].

### 2.3. Feature Extraction

**Figure 2:**
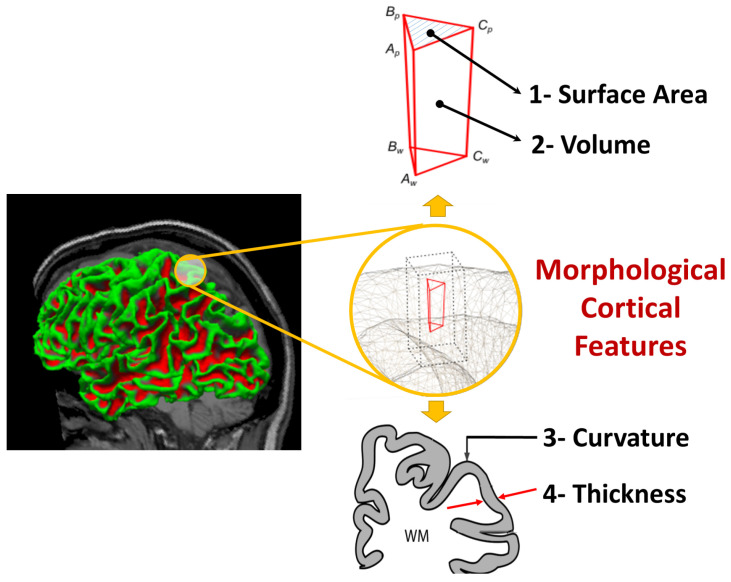
Morphological features extracted from brain surfaces by freesurfer.

There are two outputs of FreeSurfer, which are (i) a set of volumes for each subject describing each step of the pipeline (normalization, skull stripping and segmentation) as shown in Figure 1, and (ii) surfaces parcellated to DK atlas and containing the morphological features values at each point on a predefined mesh grid created on the brain, as shown in Figure 2.

In this study, we utilized the following morphological features to represent the brain of each subject: (i) surface area ($S_{a}$), (ii) volume (*V*), (iii) thickness (Th), and (iv) curvature (*c*) (see Figure 2). It is worth noting that Th is calculated as the closest distance from the gray/white matter boundary to the gray/CSF boundary at each vertex on the tessellated surface [67], while *c* is measured as the average of the reciprocal of the principal radii [57].

**Figure 3:**
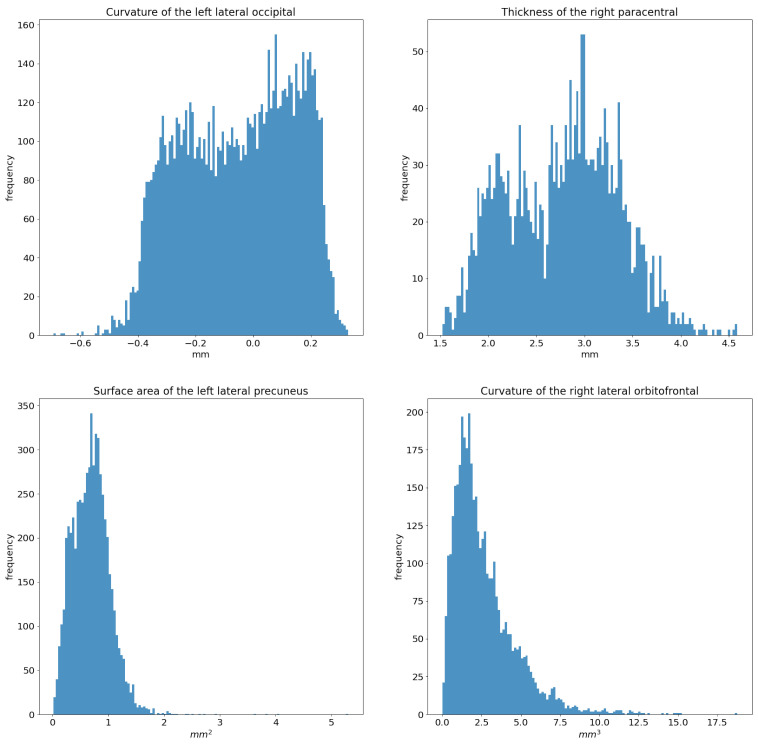
Distribution of the $S_{a}$ values within different brain regions.

For each of those features, we calculated the median value (MV), inter-quartile range (IQR), and MV±IQR within each brain region parcellated to the DK atlas. There are two reasons behind choosing MV−IQR and MV+IQR to represent each morphological feature of each brain region: (i) the distribution of morphological features’ values within each brain region is not necessary Gaussian as it is shown in Figure 3, and (ii) to include lower and upper bound that each morphological feature can possess within a specific brain region while excluding the outliers.

DK atlas parcellates the brain into 68 brain regions; 34 brain regions on the left hemisphere, and 34 brain regions on the right hemisphere. Therefore, each subject is represented by a vector of 68 brain regions × 4 features × 2 = 544 elements of a feature vector.

ABIDE I comprises 17 different sites with total number of 1112 subjects after performing quality control, and removing all subjects with bad brain segmentation, at which the data were collected. Thus, the data are heterogeneous, and it is invalid to assume blocking for all confounding variables while working on the whole dataset. Therefore, we proposed two exclusion criteria: (i) exclusion criterion for subjects, and (ii) exclusion criterion for sites.

The exclusion criterion for subjects is simply done by removing the subjects with missing feature values. The exclusion criterion for sites depends on how balanced each site is. In other words, after applying subjects’ excluding criterion, if we find a site where the ratio ASD:TD, or its reciprocal TD:ASD, exceeds 0.6, we discard that site.There is a trade-off between removing all the subjects of the unbalanced sites, or raise the unbalanced threshold. Empirically, we found that 0.6 would be a reasonable ratio, given that we utilize balanced accuracy score to evaluate the system performance, to assume balance and include as many sites as possible in the study.

The rationale behind the site’s exclusion criterion is to avoid including too many subjects of one class that have been collected with certain criterion without having their corresponding subjects from the other class that possess the same collection criterion, i.e., trying to avoid introducing more heterogeneity due to the subject’s exclusion criterion.

Table 1 shows the summary statistics of the data set after applying both exclusion criteria, subject’s exclusion criteria and site exclusion criteria. Total number of five sites have been discarded, which are: KKI, SDSU, NYU, SBL, and USM, representing a total of 305 subjects. Over the whole data set, there is no statistically significant difference between ASD and TD group (chi=−0.0271,p=0.869).

Furthermore, there is no statistically significant difference between the age of each group (t=−0.5438, p=0.5867). However, there is a statistically significant difference between the gender within each group; for TD group, chi-square test was conducted over the gender distribution $\left(\chi^{2}=84.188,\;p<0.001\right)$, and for the ASD group, the chi-square test was conducted over the gender distribution ($\chi^{2}=94.010$, p<0.001). At the end of this step, a data matrix is created as follows:

$$D=\left[\begin{array}{cccc} f_{1,1} & f_{1,2} & \cdots & f_{1,544}\\ f_{2,1} & f_{2,2} & \cdots & f_{2,544}\\ \vdots & \vdots & \ddots & \vdots \\ f_{664,1} & f_{664,2} & \cdots & f_{664,544} \end{array}\right],\;\;\;\;\;\;\;\;\;\;\;\;y=\left[\begin{array}{c} y_{1}\\ y_{2}\\ y_{3}\\ \vdots \\ y_{664} \end{array}\right]\;$$

where *D* is the data matrix with size 664 subjects ×544 features; each row represents the feature vector of a specific subject. $f_{i,j}$ denoted the feature value *j* of subject *i*, and $y_{i}$ denoted the diagnosis of subject *i*. It is worth mentioning that *D* is the data matrix for the global model.

For the local model, we created 12 data matrices ($D_{L}$) such that $D_{L}=\left\{d_{t}:d_{t}\in R^{M,544}\;\&\;1\leq t\leq 12\right\}$ each corresponding to one of the sites; $d_{t}$ denotes the data matrix corresponding to site *t*. Each $d_{t}$ has the size of M×544 such that *M* denotes the number of subjects within site t; sequentially, $y_{t}$ denotes the diagnosis vector corresponding to site *t*, and $y_{L}$ denotes the set containing all the $y_{t}$ for all sites.

$$d_{t}=\left[\begin{array}{cccc} f_{1,1} & f_{1,2} & \cdots & f_{1,544}\\ f_{2,1} & f_{2,2} & \cdots & f_{2,544}\\ \vdots & \vdots & \ddots & \vdots \\ f_{M,1} & f_{M,2} & \cdots & f_{M,544} \end{array}\right],\;\;\;\;\;\;\;\;\;\;\;\;y_{t}=\left[\begin{array}{c} y_{1}\\ y_{2}\\ y_{3}\\ \vdots \\ y_{M} \end{array}\right],\;\;\;\;\;\;\;\;\;\;\;\;D_{L}=\left[\begin{array}{c} d_{1}\\ d_{2}\\ d_{3}\\ \vdots \\ d_{12} \end{array}\right],\;\;\;\;\;\;\;\;\;\;\;\;y_{L}=\left[\begin{array}{c} y_{1}\\ y_{2}\\ y_{3}\\ \vdots \\ y_{12} \end{array}\right]$$

### 2.4. Feature Adjustment & Normalization

As it has been mentioned in the literature that there is an effect of age on ASD brain morphology [68], morphological features have been adjusted, for the effect of both age and sex, in the proposed work. Adjusted metrics of regional *V* and $S_{a}$ were calculated using cortical growth curves from Coupé et al. [69]. Denote by $V_{s}\left(a\right)$ the mean volume of cortical grey matter in individuals of sex *s* and age *a*. Then each regional volume $V_{r}$ is replaced by its age-relative, adjusted metric $V_{r}^{\prime }=V_{r}/V_{s}\left(a\right)$. Similarly, each regional surface area $S_{r}$ is converted to an adjusted metric $S_{r}^{\prime }=S_{r}/V_{s}{\left(a\right)}^{2/3}$ [69].

The feature vector corresponding to every subject contains the MV−IQR and MV+IQR of each morphological feature for every region. We consider MV−IQR and MV+IQR to be the lower bound, and the upper bound of every morphological feature for every brain region respectively. Morphological features don’t share the same units of measurement; for instance, surface area is measured in ${\mathrm{mm}}^{2}$, while *V* is measured in ${\mathrm{mm}}^{3}$. Consequently, we anticipate having different ranges of values, which might adversely affect the performance of the classifiers [70].

In this study, we utilized minimum–maximum normalization between 0 to 1 as it is one of the most common normalization methods used for biomedical data [71]. Consequently, each column in the data matrix *D* is normalized between 0 to 1 using the Equation (1).

(1) $${\tilde{f}}_{i,j}=\frac{f_{i,j}-\min_{i}\left\{f_{i,j}\right\}}{\max_{i}\left\{f_{i,j}\right\}-\min_{i}\left\{f_{i,j}\right\}}$$

where ${\tilde{f}}_{i,j}$ and $f_{i,j}$ denote the normalized feature value *j* and the original feature value *j* corresponding to the subject *i*, and $\min\left(f_{j}\right)$ and $\max\left(f_{j}\right)$ correspond to minimum and maximum values of the feature vector *j* respectively. The output normalized matrix is denoted by $D_{n}$ for the global model, and $d_{tn}$ for the local model where 1≤t≤12.

### 2.5. Building Neuro-Atlas

To implement a Computer-Aided Diagnosis System (CAD) for accurate diagnosis of autism, we have to use a neuro-atlas tailored to the specific developmental patterns of the brain in autism. Unfortunately, there is no general purpose brain atlas in the literature that we can use in our CAD system; thus, developing an atlas for autistic subjects that shows the areas and imaging markers that are associated with autism is the main motivation behind this work. To achieve this goal, we used the modern tools of machine learning (e.g., Recursive Feature Elimination via Cross Validation (RFECV)) to select the most significant features and their corresponding areas that are correlated with autism spectrum disorder.

**Figure 4:**
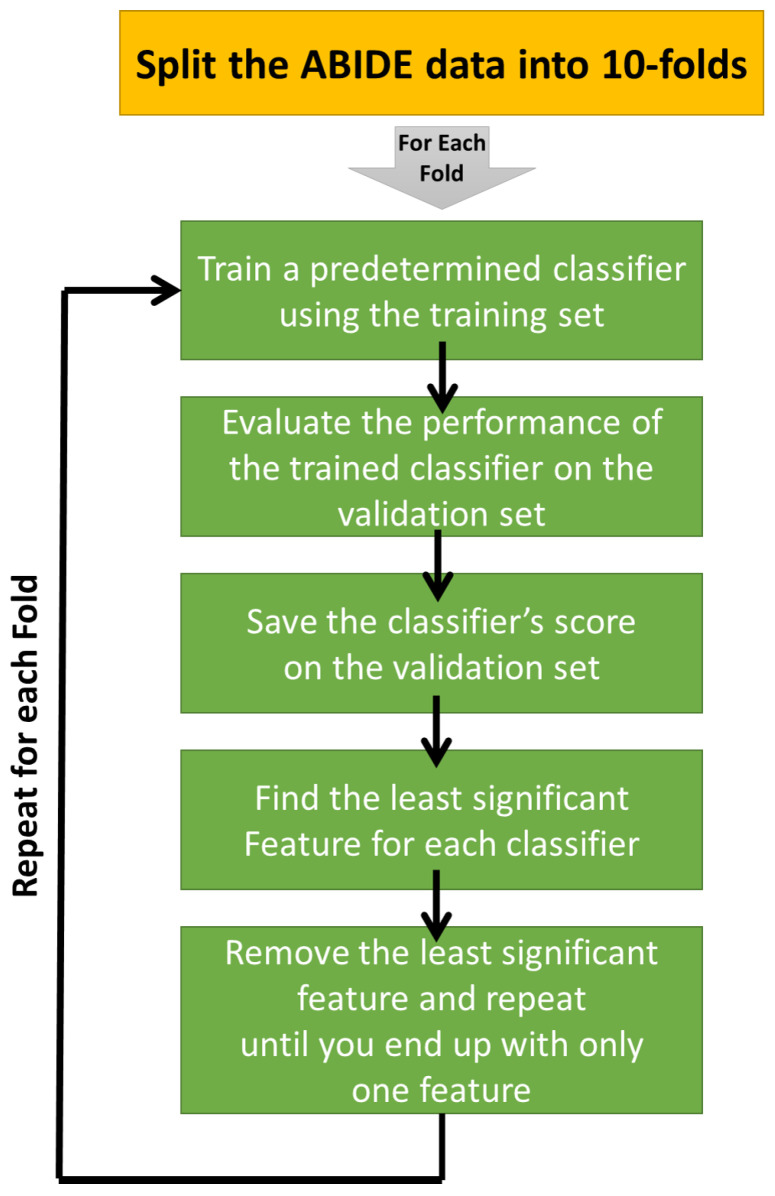
The flowchart of The RFECV algorithm.

Since RFECV is one of supervised feature selection algorithm, we have to split the data into *k*-folds, k=10 in our case (as shown on Figure 4, and for each fold (i) train a predetermined classifier using the training set, (ii) evaluate the performance of the trained classifier on the validation set, (iii) save the classifier’s score on the validation set, (iv) find the least significant feature according to the trained classifier, (v) remove the least significant feature from the model, and (vi) repeat the whole process until you end up with only one feature.

Again, repeat the whole process for each fold, calculate the average performance of the k−fold cross-validation (CV) when: using all features to train the classifier; using all features but one, and so forth, to the point of classification on a single feature. Find the number of the features at which the classifier has the maximum performance score, assuming it is $N_{f}$ features. $N_{f}$ is the optimum number of features to be selected.

Perform the whole algorithm again over all the subjects to find the most $N_{f}$ significant features. The algorithm is discussed in detail in Algorithm 1. For further details regarding the algorithm and its implementation, the reader is suggested to read Guyon et al. [72] and Pedregosa et al. [73], respectively.

To build a neuro-atlas for autism, ABIDE I dataset and RFECV are utilized to select those significant brain regions along with their morphological features. For both the local model and the global model, RFECV is run with four different classifier architectures, which are RF, least absolute shrinkage and selection operator (LASSO), RIDGE regression (RIDGE), and SVM with linear kernel, resulting in four different models.

Those four models represent two major categories of features’ sets: (i) A feature set that forms a feature space, where the subjects are non-linearly separable as much as possible, and (ii) A feature set that forms a feature space, where the subjects are linearly separable as much as possible. The first category corresponds to the features’ set selected by RFECV+RF, and the second category corresponds to the features’ sets selected by RFECV+LASSO, RFECV+RIDGE, RFECV+SVM.

Each of the RFECV models is performed with 10-fold CV; such that we iterate over all the 544 features, removing one feature at a time, perform 10-fold CV on the current sample, and calculate the average balanced accuracy score. The balanced accuracy score was introduced in 2010 to solve the optimistic estimate occurs when a biased classifier is tested on an imbalanced dataset [74]. The balanced accuracy score is defined by Equation (2):

(2) $$score=\frac{1}{2}\left(\frac{TP}{Pos}+\frac{TN}{Neg}\right)$$

where score denotes the balanced accuracy score, TP denotes the true positive classified by the model, Pos denotes the total number of positive cases in the sample, TN denotes the true negatives classified by the model, and Neg is the total number of negative cases in the sample.
**Algorithm 1:** RFECV
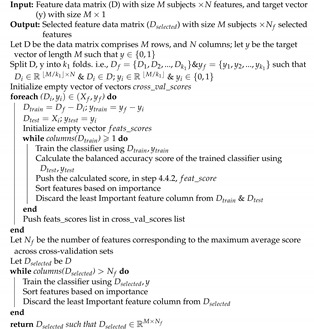


For the site-based model, RFECV is performed on each site separately. The selected set of features is then extracted for each site to have a new data matrix with number of columns less than or equal to the original number of columns. For the global model, RFECV is performed only one time on the normalized data matrix $D_{n}$, and the selected set of features is calculated and then extracted from $D_{n}$. At this point, we assume that the selected features from each site are the imaging markers candidate for ASD subjects collected from that site i.e., local imaging markers, while the selected features from $D_{n}$ in the global model are the global markers candidates that define the ASD subjects in the whole dataset.

In the case of the global model, the input to the RFECV step is $D_{n}$ and the output is $S_{n}$ where the size of $S_{n}$ is 664×M such that *M* ≤ 554. In the case of the site-based model, the input to the RFECV step is 12 $d_{n}$ (normalized data matrices of each site), and the output is $s_{kn}$ where the size of $s_{kn}$ is N×M such that *N* is the number of subjects within site *k*, and *M* ≤ 544.

Eventually, a global neuro-atlas is created using the whole data set, and a local neuro-atlas is created for each site. We claim that the globla neuro-atlas, as well as, the local neuro-atlases can be used as a guide for future analysis of ASD or ABIDE I dataset.

### 2.6. ML Classifiers

Having the imaging markers candidates, they should be placed under test to see how good they are at separating the two classes. A set of eight different ML classifiers representing both linear and non-linear hypotheses is selected to test the local, and the global imaging markers candidates. The utilized eight ML classifiers are split into two main divisions: (i) linear classifiers, and (ii) non-linear classifiers. The linear classifiers set comprises LR, LSVM, and passive aggressive. The non-linear classifiers set comprises RF, SVM with radial basis function (SVM-RBF), eXtra Gradient Boost trees (xgboost), Gaussian Naive Bayes (GNB), and neural network (NN) shallow and deep.

To optimize the hyper-parameters of each classifier, the data matrix is reduced accordingly to the results of RFECV algorithm. The data is split five fold. For each of the predefined classifiers, the hyper-parameters and their ranges, where the search will be conducted, are defined in the Supplementary Material S2, and then a nested for-loop for each classifier. For each hyper-parameter value of that classifier, a five-fold CV is performed, and the results of each fold are saved.

Eventually, the hyper-parameters that corresponds to the maximum CV average score is saved as the optimum parameters. The results of the highest performed classifiers are saved with their hyper-parameter values. The performance metric for each classifier is set to the balanced accuracy score. The detailed algorithm is shown in Algorithm 2.**Algorithm 2:** ML-train with hyper-parameter optimization.
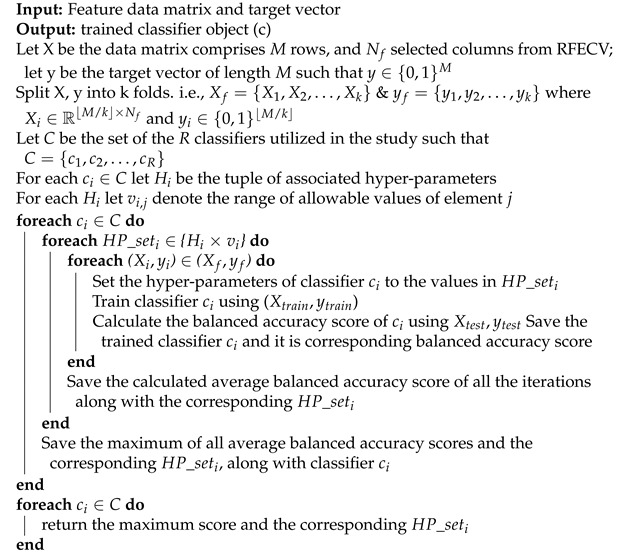


### 2.7. Personalized Diagnosis

We propose a personalized map per subject to show the affected brain regions and to gage the probability of a diagnostic difference when comparing autistic individuals to control. We define a personalized map as a set of scores associated with a set of features denoting the importance of a particular feature in diagnosing a subject as TD or ASD. In a previous work [13], we created a personalized map for ABIDE I dataset using both fMRI and sMRI features. However, in the proposed work, we are introducing a personalized map for ABIDE I dataset using only sMRI morphological features. The motivation behind creating the personalized map for only the sMRI morphological features is the high performance of the proposed pipeline.

A personalized map is created for each local model. The personalized maps are easily created with classifiers that either associate weights to the input features, such as linear classifiers, or place the input features in a tree schematic that denotes the importance of each feature based on the level of the feature. However, since NN is the used classifier for the local models, it is difficult to determine which input feature contributes the most to the classification decision and which input feature contributes the least. 

Local interpretable model-agnostic explanations (LIME) [75] is a novel explanation technique that explains the prediction of any classifier. The main idea behind LIME is that it builds linear models around the predictions of an opaque model to explain it. LIME is used to explain the classification decisions made by four different local models We have used LIME to explain the decisions of four local models on two random subjects within each of the four sites. Afterward, we visualize the scores representing the contribution of each feature in the classification decision.

## 3. Results

In this section, the results for each site are demonstrated in terms of (i) autism imaging local markers (AILM) for each site, and (ii) the balanced accuracy score of the trained ML when using the AILM. The balanced accuracy scores of the trained ML models are meant to validate the AILM selected via one of the proposed feature selection models (RFECV+lg1, RFECV+lg2, RFECV+svm, RFECV+rf). In order to measure the significance of our findings, we compare the accuracy of the proposed pipeline in classifying ASD to the results of Katuwal et al. [76] study. Katuwal et al. utilized ABIDE I dataset to provide a site-based diagnosis using RF, SVM, and Gradient Boosting Machine (GBM) classifiers.

In addition to the results of the local model, we applied the proposed on the whole dataset to find, what we have called, autism imaging global markers (AIGM). Similar to local model, AIGM are put to test to study how discrimintive they are at classifying ASD. However, for the global model we do not only compare the results with another study, but also, we compare the results with the proposed model without performing feature selection i.e., using 544 features.

Therefore, for the global model the two comparisons performed are (i) compare the performance, utilizing balanced accuracy score, of the proposed model to the performance of the same model while excluding the feature selection step, and (ii) comparing to the results of Sabuncu et al. [77]. Sabuncu et al. performed univariate and multivariate methods to classify ASD subjects within ABIDE I dataset. The authors performed five-fold cross validation and used SVM, Neighborhood approximation forest (NAF), which is a variant of random forest, and relevance voxel machine (RVM) classifiers.

Sabuncu et al. used a different set of features as well; however, the highest classification accuracy was achieved using the cortical thickness values sampled onto the fsaverage5 template, provided by Freesurfer, and smoothed on the surface with an approximate Gaussian kernel of a full-width-half-max (FWHM) of 5 mm.

**Figure 5:**
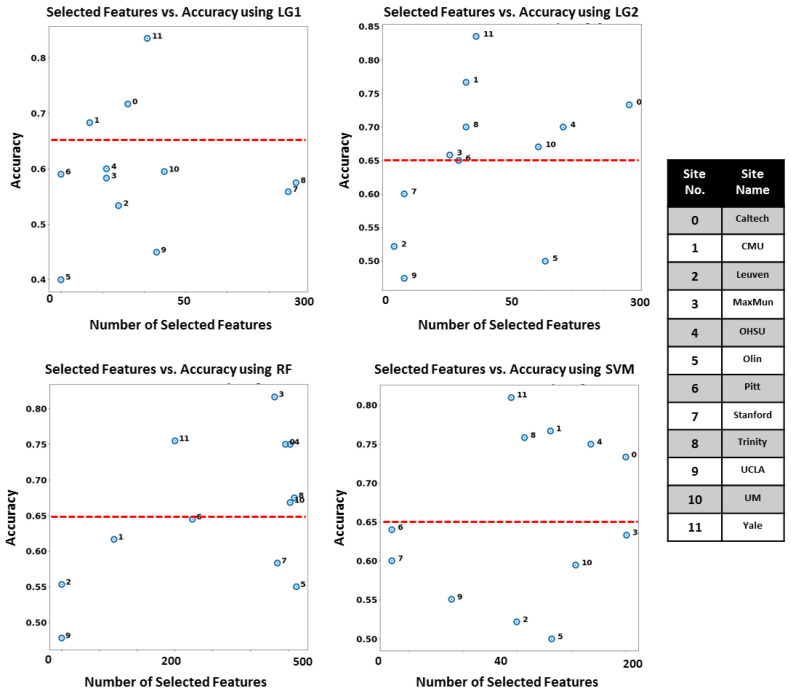
Number of selected features vs. the maximum balanced accuracy score achieved using these features when applying RFECV using the four core classifiers, using the local model.

**Autism Local Neuro-Atlases:** RFECV with the four classifiers is applied on each independent site, and the set of features corresponding to the maximum balanced accuracy score is selected. To compress the data visualization into four subplots similar to the case of the global model, we visualize only the optimum number of features selected by each RFECV model along with the highest achieved balanced accuracy score. Figure 5 demonstrates the number of selected features corresponds to the maximum balanced accuracy score achieved when utilizing each RFECV model on every site of the dataset.

**ML local models:** almost perfect cross-validation results are achieved when using the local-atlases selected by RFECV+lg2, as it showed the highest performance in case of the global model, as well as in case of sites-based model, the highest number of sites with balanced accuracy score above 0.65 is achieved with RFECV+lg2 algorithm. Results are compared with Katuwal et al. [76] in Table 2. The proposed pipeline has achieved better accuracy as shown in Table 2. Although Katuwal et al. selected, for some sites, a smaller number of features, the proposed pipeline has outperformed their method in terms of accuracy.

**Figure 6:**
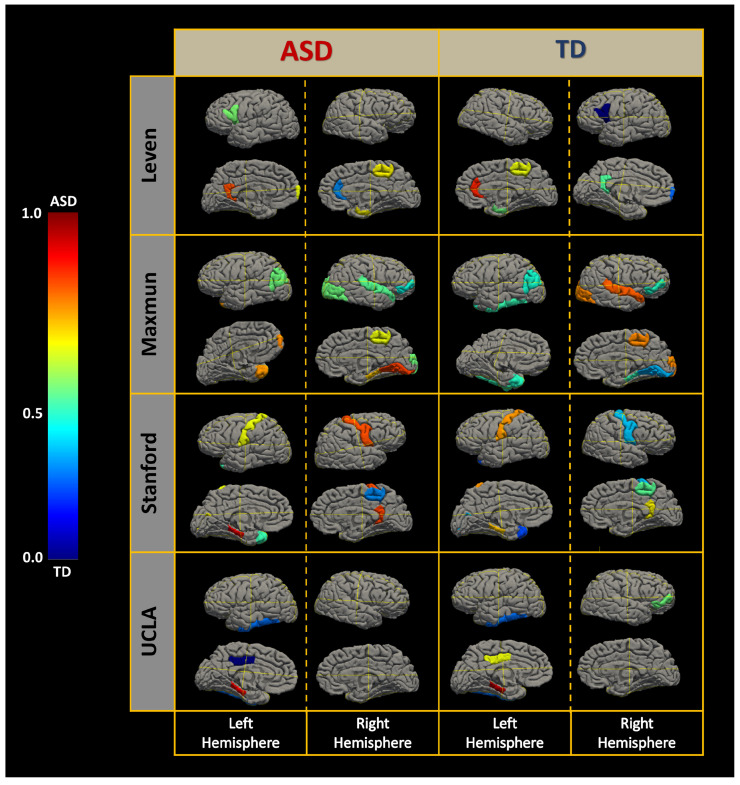
Personalized diagnosis.

**Personalized diagnosis:**Figure 6 visualizes the results of applying LIME on eight different subjects from four different sites. brain regions with gray color represents brain regions that are not selected as candidates of the local neuro-atlas for that site. Brain regions with deep blue color contribute heavily toward classifying the subject to be ASD and it gets milder as the color moves from blue towards green.

On the other hand, brain regions with a deep red color contribute heavily toward classifying the subject to be TD and it gets milder as the color moves from the red color to the green color. All the subjects shown in Figure 6 are correctly classified by their corresponding local models.

Figure 6 indicates that the local models classify subjects based on the majority voting of the classifications per brain region. However, we cannot assume that using each brain region solely for classification and aggregating their decisions would yield the same results. We assume that each of the local neur-atlas creates a multidimensional space at which the highest possible accuracy of classification of ASD is achieved, and it is hard to achieve the same accuracy by utilizing each feature independently from the others.

**Figure 7:**
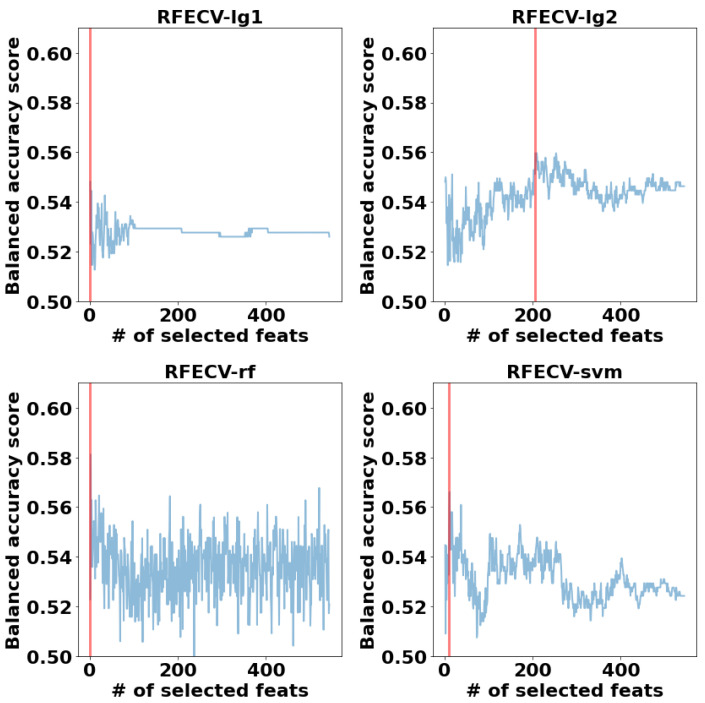
The number of selected features vs. the balanced accuracy score when applying RFECV with different classifiers. The red vertical line labels the number of features corresponding to the maximum balanced accuracy score.

**Autism Global Neuro-Atlas:** The high accuracy obtained using the local model has encourage us to try to find a global model. To achieve this goal, we performed the same feature selection criteria on the whole data set to determine the autism global neuro-atlas. Figure 7 demonstrates the optimum number of the selected features using each of the four RFECV models. The number of features selected on each trial is represented on the horizontal axis. On the other hand, the average performance of the five-folds, when RFECV utilized that specific number of features, is represented on the vertical axis. The optimum number of features corresponding to the maximum average balanced accuracy score is denoted by a vertical red line.

The features set containing that optimum number of features is then utilized to train the ML models of the following step in the pipeline. The results demonstrated in Figure 7 are as follow. RFECV+lg1 selected only one feature, RFECV+lg2 selected 207 features, RFECV+rf selected 2 features, and RFECV+lSVM selected 11 features. It is worth noting that all the selected features are already included in the RFECV+lg2 set. The selected features’ set, representing the global neuro-atlas, is found in Supplementary Materials S1.

**Figure 8:**
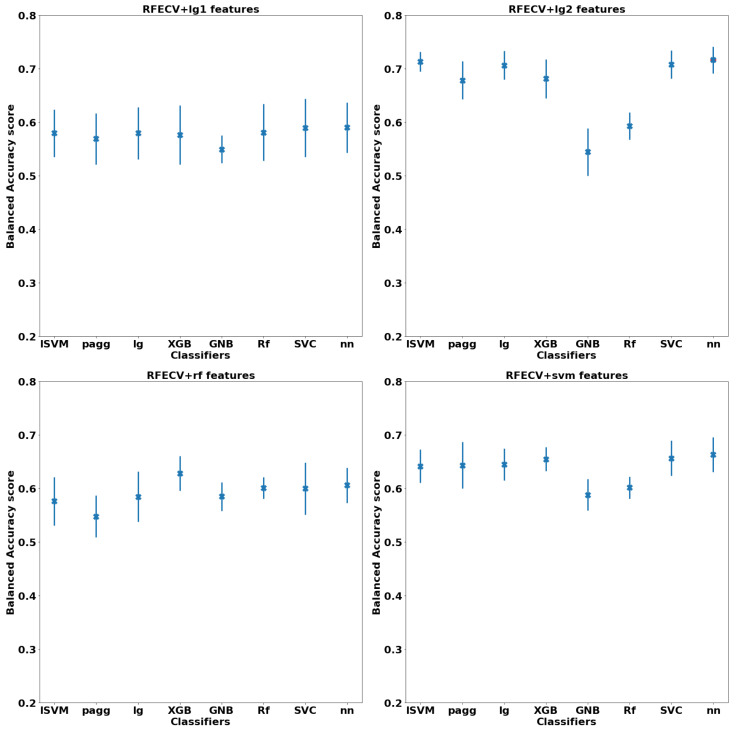
The highest testing balanced accuracy score ± standard deviation achieved by each of the optimized classifiers with applying RFECV with the core classifiers. The red dot labels the classifiers with the highest mean testing accuracy over the five-folds cross-validation.

**ML global model:** the balanced accuracy scored is calculated for each of the four selected features’ set utilizing all of the eight classifiers. Figure 8 demonstrates the mean and std. deviation over the five-fold cross-validation when using the optimum hyper-parameters of each classifier. The highest balanced accuracy score, marked as red dot on the figure, is achieved when using the features (global neuro-atlas) selected with lg2 classifier and utilizing NN (0.716±0.024) The optimum hyper-parameters used for NN are as follows: loss function is Cross-Entropy, solver for weight optimization is stochastic gradient descent, learning rate is set to be adaptive, l2 penalty (regularization term) is 0.0001, activation function is hyperbolic tangent function (Tanh).

**Figure 9:**
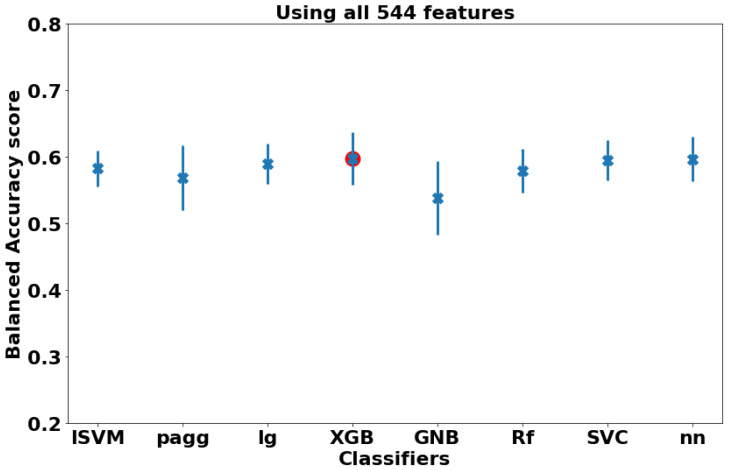
The highest testing balanced accuracy score, plus or minus one standard deviation, achieved by each of the optimized classifiers without applying any feature selection algorithms.The red point labels the classifiers achieving the highest performance.

Similarly, for the null hypothesis, the same hyper-parameter optimization has been carried out for the eight classifiers, except that all the 544 were utilized without performing any feature selection. Figure 9 demonstrates the balanced accuracy score calculated for each of the optimized eight classifiers. The highest achieved score, marked as a red dot on the figure, is achieved with XGB classifier (0.597±0.04). A summary of the global model results is shown in Table 3.

## 4. Discussion

In this section, we discuss the global and local neuro-atlases. We cover the common findings between the proposed study and previous literature. We focus on two aims: (i) creating a discriminative model using a set of morphological features extracted from sMRI volumes of the brain of ASD and TD subjects, and (ii) defining global and local neuro-atlases that can be used to define ASD; consequently, those atlases can be used to understand the underling neurophysiology of the disorder.

We define a discriminative model as a ML classifier that is trained using a set of features that best discriminate ASD and TD in the current dataset. In this study, we introduced two different models: (i) the global model that aims to classify ASD and TD in a heterogeneous dataset, (ii) the site-based model/local model that aims to classify ASD and TD in a less heterogeneous dataset based on demography.

### 4.1. Analysis of the Relation between Global and Local Models

The RFECV algorithm is performed on the data matrix using four different classifiers: Lg1, Lg2, SVM, and RF. Each model selects the set of features that maximizes the classification’s balanced accuracy score via 10-fold CV process. Those sets of features are considerd as local neuro-atlases for ASD within each site. The algorithm is performed only one time in case of the global model, while it is performed 12 times on each site in case of the local model.

The main motivation behind estimating a global model is the promising results of the local model. The discriminative set of features is determined at the ML step where a set of linear and non-linear classifiers are trained on the selected features set. The set of features corresponding to the maximum balanced accuracy score via five-fold CV is said to be the discriminative feature set (neuro-atlas) for either the global model, or for a specific site.

In the case of global model, RFECV+lg2 with 207 morphological features corresponding to a balanced accuracy score of (0.715±0.024) is set to be the model that has selected the discriminative set of features. The 207 features can be found in Supplementary Materials 1. Table 4 shows the RFECV model corresponding to the maximum classification balanced accuracy score of a five-fold CV process, along with the size of the selected features’ set and the number of common features with the global model.

Common features are anticipated between the global model and local models since the global model can be thought of as something “similar” to the aggregation of the local models. However, this raises the question about whether any of the mutual features are repeated within multiple sites as well as the global model.

Across all the 544 features, the highest number of selections to be given for a feature is five times i.e., a feature is selected by the global model and four different local models. We selected the features that are nominated by the global model as well as at least three different local models in order to study them to be candidate imaging markers. Table 5 demonstrates the morphological features, the hemisphere, and the brain regions with the highest frequency of selection among sites in a decreasing order.

**Figure 10:**
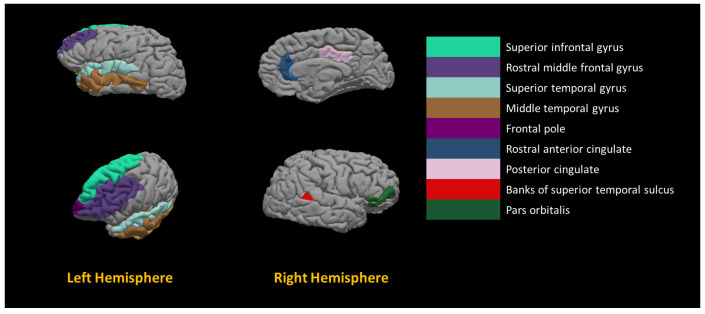
Visualization of the most frequent brain regions to be selected by RFECV+LG2.

A total number of 16 features met the aforementioned criteria. Among those 16 features, the distribution of the morphology is as follows: (i) surface with five occurrences, (ii) curvature with four occurrences, (iii) volume with four occurrences, and (iv) thickness with three occurrences. There are eight brain regions from the left hemisphere and eight brain regions from the right hemisphere. The most frequent brain regions among the 16 most common are the Middle Temporal Gyrus and Transverse Temporal Gyrus. The unique selected brain regions are shown on brain template image in Figure 10.

### 4.2. Detected ASD Neurocircuits

The development of a CAD system for the early diagnosis of autism must include central features that correspond to the effect of the increasing neuropil within a brain region. Through these experimental approaches, structural MRI parameters related to the expanding neuropil are relevant to defining ASD neurocircuits. The effect of the local diagnosis identifies ASD related brain regions, which fit into the Research Domain Criteria (RDoC) neural circuits and are similar circuits predictive of ASD diagnosis at 24 months. Neurocircuits detected in the proposed work is shown in Table 6.

RDoC-defined language and social neural circuits are overly represented in the morphological data. Middle Temporal Gyrus has been found to be associated with autism volume of left Middle Temporal Gyrus [79,80], functional connectivity of left and right Middle Temporal Gyrus [78,81]. Transverse Temporal Gyrus has been found to possess significant difference between ASD and TD in terms of magnetic mismatch field evoked by voice stimuli in 3- to 5- year-old subjects [82].

Superior Temporal Gyrus is found to be possess a greater gray matter volume in ASD subjects who show drive to assess or construct rule-based systems [83], a diminished functional connectivity between cerebellum in ASD subjects of ABIDE I dataset [84], other functional connectivity differences, occurred in superior temporal gyrus, between ASD and TD subjects in three age cohorts <12, 12–19, and >20 years old [85], more different morphological alterations have been reported for superior temporal gyrus in different studies [79,86,87,88]. Banks of Superior Temporal Sulcus are found to demonstrate less developmental trajectory of the surface area [89], and an increased thickness of the right Banks of Superior Temporal Sulcus [90].

Reward learning, attentional, social, and executive function RDoC defined neural circuits are also impacted and classify individuals as ASD or TD. The frontal pole has been found to have a decreased thickness in the left hemisphere for the ASD subjects in the age of (9.2 ± 2.1 years) [91]. Rostral Anterior Cingulate has found to demonstrate abnormally increased activation on specific visual tasks in fMRI and reduced fraction anisotropy in the white matter [92], and cortical thickness alteration [45].

Rostral Middle Frontal Gyrus is found to be the most important feature for classification in a dataset of children older than 6 years old [93], the volume of rostral middle frontal was found to be the only statistically significant difference between ASD and TD group with the age of 33 ± 9.1 year-old [94].

Lateral Occipital Sulcus is found to have a negative slope of relationship between local gyrification index and age greater in ASD than TD; moreover, a negative correlation between the local gyrification index and cortical thickness if found to be less in magnitude in ASD than TD [95].

A study focusing on finding brain regions that can be targeted by noninvasive brain stimulation (NIBS), for ASD treatment labeled the Lateral Occipital Cortex as a potential target for NIBS [96]. Similarly, the social function associated circuits involving the Posterior Cingulate Cortex are found to possess irregularly distributed neurons, and there is an increased density of neuron in the underlying white matter in the same region [97].

Furthermore, in an fMRI study, the ASD group showed weaker connectivity between the posterior cingulate cortex and superior frontal gyrus, stronger connectivity between posterior cingulate cortex and both the right temporal lobe and right Para hippocampal gyrus. Poorer social functioning in the ASD group was correlated with weaker connectivity between the posterior cingulate cortex and the superior frontal gyrus [98].

Banks of Superior Temporal Sulcus was found to demonstrate less developmental trajectory of the surface area [89] and an increased thickness of the right Banks of Superior Temporal Sulcus [90].

The proposed pipeline is anticipated to achieve better results than those in the literature because of the way that the morphological feature values are aggregated is less prone to outliers, RFECV implementation with more than one classifier to cover as many assumptions on the relationship between the features and the target as possible while selecting the features, performing hyper-parameter optimization using grid search on eight classifiers to achieve the optimum results given the selected set of features. All the codes utilized in the proposed pipeline implementation are available upon request.

## 5. Limitations and Conclusions

In the proposed study, we designed and implemented a ML pipeline to identify neuro-atlas of ASD. The proposed pipeline includes data preprocessing, feature extraction, feature normalization and age adjustment, feature selection via four different RFECV models, and classification using hyper-parameter optimization of linear and non-linear machine learning models.

The most discriminative set of features (neuro-atlases) is formed using RFECV+lg2 model. The resultant neuro-atlases are used to train a set of linear, and non-linear classifiers. The highest balanced accuracy score is achieved by NN for both the global model and the local model with average balanced accuracy score of 71.6±2% and 97±2%, respectively. The most common features among the global model and each site of the local model are then analyzed to create ASD neurocircuits.

The two main steps that helped in achieving the high results are: (i) Building neuro-atlases via RFECV and (ii) Hyperparameter optimization of the classifiers. We could not ignore the threat of overfitting especially for the sites that achieved 100% accuracy with big number of features compared to the number of subjects within those sites. Nevertheless, we followed every machine learning experimentation best practices to the best of our knowledge.

We split out data into five folds, training with four folds and testing with the rest. We evaluated models based on the average performance of the ML model on the five splits. We achieved 100% accuracy. However, and as it was stated in the discussion section, we believe that within each of the ABIDE I dataset sites, there is some sort of homogeneity between the subjects. This homogeneity is not only demographic but rather homogeneity of the disorder itself, and therefore we believe that, for the case of Caltech, and other sites, a couple of hundred of cortical morphological features were able to capture all of the autism patterns within those specific sites.

Consequently, this is why we decided to perform the neuroatlas analysis to study whether there are any connections, i.e., neurocircuits, within those couple of hundreds of cortical features that were able to capture 100% of the variance within those specific sites. Further validation should be done, and we are looking forward to this in our next study.

The overall structural mapping of cognition and behaviors to distinct neuroanatomical and functional linked neural circuits is more likely to not only diagnosed but map a cluster of ASD individuals whose behaviors and characteristics are more similar than different. The proposed pipeline is anticipated to achieve better results than those in the literature because of the way that the morphological feature values are aggregated is less prone to outliers.

RFECV implementation with more than one classifier will also cover as many assumptions on the relationship between the features and the target as possible while selecting the features while performing hyper-parameter optimization using grid search on eight classifiers to achieve the optimum results given the selected set of features. All the codes utilized in the proposed pipeline implementation are available upon request.

Neuroimaging is an attractive non-invasive technology to facilitate the definition of relationship between genes, environment, and behaviors in ASD. While this study’s numbers, design, mitigation of age/sex, pre-processing, etc. lend credence to these results, the truth is that the use of sMRI and fMRI data is still a challenge as large datasets from typically developed children from infancy through 8 years of age are still lacking. The current sample size does identify brain regions implicated infants who are at high risk for ASD suggesting that this approach is scalable for use in larger more heterogeneous groups of ASD populations.

The higher accuracy of ASD classification in this study also reinforces this hypothesis. Ultimately, the proposed system should provide a complete map explaining what linked brain regions are affected, to what extent impairments are more severe and could, thereby, be very useful to a treating physician/provider from a clinical point of view.

We hypothesize the difference in the balanced accuracy score of the global model and the local model is due to the high heterogeneity of the disorder. This hypothesis is based on the number of common features among the sites of the local model, as well as the number of features selected for both the global and local model. Consequently, for future work, we are planning to do the following: (i) incorporate the ABIDE II dataset along with ABIDE I and (ii) partition subjects based on behavioral traits in order to subdivide ASD into more types of “homogeneous ASD” where subjects share more traits.

Eventually, we proposed a personalized diagnosis method in which we describe the phenotype of each subject in terms of the local imaging markers values. The outcome of this step is a personalized model that describe the affected brain regions, which made the classifier decide a specific subject to be ASD. We hypothesized that the affected brain region, giving their feature values, might be correlated with a brain physiological anomaly that might be causing a specific autistic behavior. Thus, by recognizing those affected brain regions, a personalized treatment can be assigned for each subject to help with autistic traits moderation.

## Figures and Tables

**Table 1:** ABIDE data phenotypical information summary after sites’ preprocessing.

Site	Total	ASD		TD	
		n	Age (Min, Max)	n	Age (Min, Max)
Caltech	37	19	(17.5, 55.4)	18	(17, 56.2)
CMU	27	14	(19, 39)	13	(20, 40)
Leuven	63	29	(12.1, 32)	34	(12.2, 29)
MaxMun	51	23	(7, 58)	28	(7, 46)
OHSU	26	12	(8, 15.2)	14	(8.2, 11.9)
Olin	34	19	(11, 24)	15	(10, 23)
Pitt	56	29	(9.33, 35.2)	27	(9.4, 33.2)
Stanford	38	19	(7.5, 12.9)	19	(7.7, 12.4)
Trinity	47	22	(12, 23)	25	(12, 25.6)
UCLA	95	53	(8.36, 17.94)	42	(9.2, 17.7)
UM	134	61	(8.5, 18.6)	73	(8.2, 28.8)
Yale	56	28	(7, 17.7)	28	(7.6, 17.8)
Total	664	328		336	

**Table 2:** Comparison between the proposed pipeline and previous results from the literature.

Site	Katuwal et al. [76] Results		Proposed Pipeline	
	Number of Selected Features	Accuracy (%)	# of Selected Features	Accuracy (%)
Caltech	5	97	217	100
CMU	1	94	18	100
Leuven	-	-	20	91.5±5
MaxMun	-	-	151	97.5±1
OHSU	12	77	3	100
Olin	1	86	60	100
Pitt	-	-	16	100
Stanford	-	-	7	100
Trinity	-	-	18	100
UCLA	2	64	55	82.2±5
UM	3	72	59	97.2±1
Yale	2	75	21	100

**Table 3:** The classification accuracy score of the Sabuncu et al. study, and the proposed model with and without RFECV, along with the classifier used to achieve the score for each model and the number of features included in each model.

	Sabuncu et al. [78] Results	Proposed Pipeline without RFECV	Proposed Pipeline
Accuracy	0.59±0.02	0.597±0.04	0.716±0.02
Classifier	RVM	XGB	NN
# of features	20484	544	207

**Table 4:** Summary statistics of the selected features using the local model.

Site	# of Features	# of Mutual Features with the Global Model
Caltech	217	74
CMU	18	5
Leuven	6	4
MaxMun	14	7
OHSU	79	32
Olin	60	26
Pitt	16	8
Stanford	7	1
Trinity	18	5
UCLA	7	2
UM	54	23
Yale	21	9

**Table 5:** The most frequent morphological features and brain regions to be selected by RFECV+lg2 discriminative model.

Morphological Feature	Hemisphere	Brain Region
Curvature	Left	Middle Temporal Gyrus
Volume	Left	Middle Temporal Gyrus
Volume	Left	Transverse Temporal Gyrus
Surface area	Right	Transverse Temporal Gyrus
Curvature	Left	Frontal Pole
Curvature	Right	Rostral Anterior Cingulate
Curvature	Right	Transverse Temporal Gyrus
Thickness	Left	Middle Temporal Gyrus
Thickness	Left	Rostral Middle Frontal Gyrus
Thickness	Left	Superior Temporal Gyrus
Volume	Right	Lateral Occipital Sulcus
Volume	Right	Posterior Cingulate Cortex
Surface area	Left	Superior Frontal Gyrus
Surface area	Right	Banks of Superior Temporal Sulcus
Surface area	Right	Pars Orbitalis
Surface area	Right	Pars Orbitalis

**Table 6:** The brain regions linked to RDoC Neural Circuits, Behavioral/Cognitive Domains of the ADOS, and ASD Structural Diagnosis.

Component	RDoC Neurocircuit	ADOS Domain	Anatomical Correspondence
Restricted Interest	Reward Learning/Habit	RRB	Frontal Pole
Attention	Ventral/Dorsal Attention	Total	Rostral Middle Frontal Gyrus
			Lateral Occipital Sulcus
Language	Receptive/Expressive	SA	Middle Temporal Gyrus
			Transverse Temporal Gyrus
			Pars Orbitalis
			Superior Temporal Gyrus
			Superior Temporal Sulcus
Social	Affiliation & Attachment	SA	Frontal Pole
Social	Self Aware	SA	Superior Frontal Gyrus
			Posterior cingulate gyrus
Social	Understanding the Mental States of Others	SA	Rostral ACC
			Superior Temporal Sulcus
Executive Function	Working Memory	SA	Superior Frontal Gyrus
			Rostral Middle Frontal Gyrus
	Performance Monitoring	SA	Rostral ACC

## Data Availability

The adopted data are publicly available at https://fcon_1000.projects.nitrc.org/indi/abide/abide_I.html (accessed on 8 December 2021).

## Supplementary Materials

The following supporting information can be downloaded at: https://www.mdpi.com/article/10.3390/diagnostics12010165/s1, Supplementary Material S1: The complete definition of all sites, Supplementary Material S2: Hyper-parameters range of each classifier.
