# Supplementary material for: The Role of Structure MRI in Diagnosing Autism
Source: Diagnostics (Basel). 2022 Jan 11;12(1):165. doi: 10.3390/diagnostics12010165 (PMC8774643; doi:10.3390/diagnostics12010165)
Supplement: Supplementary file 1 [file diagnostics-12-00165-s001.zip › S1.pdf]

Supplementary Material S1: The complete definition of all sites

Site	Definition
Caltech	California Institute of Technology
CMU	Carnegie Mellon University
Leuven	University of Leuven
MaxMun	Ludwig Maximilians University Munich
OHSU	Oregon Health and Science University
Olin	Olin, Institute of Living at Hartford Hospital
Pitt	University of Pittsburgh School of Medicine
Stanford	Stanford University
Trinity	Trinity Centre for Health Sciences
UCLA	University of California, Los Angeles
UM	University of Michigan
Yale	Yale Child Study Center
