# Supplementary material for: The Role of Structure MRI in Diagnosing Autism
Source: Diagnostics (Basel). 2022 Jan 11;12(1):165. doi: 10.3390/diagnostics12010165 (PMC8774643; doi:10.3390/diagnostics12010165)
Supplement: Supplementary file 1 [file diagnostics-12-00165-s001.zip › S2.pdf]

Supplementary Material S2 Hyper-parameters range of each classifier

Classifier	Hyper-parameters	var_ame	values
3*Linear SVM	Regularization parameter	C	0.1, 1, 5, 10
	Norm used in the penalization	penalty	”l1”l2”
	Loss function	loss	hinge ßquared_inge”
2*Passive Aggressive Classifier	Regularization parameter	C	0.1, 1, 5, 10
	Number of iterations to stop after no improvement occurs	n_ter_o_hange	1, 5, 10
3*Logistic Regression	Norm used in the penalization	penalty	”l1 ”l2 ëlasticnet”
	Regularization parameter	C	0.1, 1, 5, 10
	Algorithm to use in optimization problem	solver	newton-cg ”lbfgs ”liblinear ßag ßaga”
7*Extreme Gradient Boosting Classifier	type of booster	booster	”gbtree ”gblinear ”dart”
	Learning rate	learning_ate	0.001, 0.01, 0.1, 0.3, 0.5, 1
	Minimum loss reduction required to make a further partition on a leaf node of the tree	gamma	0, 0.1, 0.5, 1, 1.5, 2, 5, 20, 50, 100
	Minimum sum of instance weight (hessian) needed in a child	min_hild_eight	0.1,0.5, 1, 5, 10
	Number of boosting rounds	n_stimators	50, 100, 200, 500, 1000
	L1 regularization term on weights	reg_lpha	0, 0.001, 0.01, 0.1, 0.5, 1, 5, 10
	L2 regularization term on weights	reg_ambda	0, 0.001, 0.01, 0.1, 0.5, 1, 5, 10
5*Random Forest	Number of trees in the forest	n_stimators	50, 100, 200, 500, 1000
	Function to measure the quality of a split	criterion	giniëntropy”
	Number of features to consider when looking for best split	max_eatures	sqrt”log2All features
	Minimum number of samples required to split an internal node	min_amples_plit	1, 2, 5, 10
	Whether to use bootstrap samples while building the tree or use the whole training set	bootstrap	True, False
5*Non-linear SVM	kernel used	kernel	poly”rbfßigmoid”
	Regularization parameter	C	0,0.1,1,5, 10
	Degree (when kernel is polynomial)	degree	2,3,4,5,6
	Kernel coefficient	gamma	scaleäuto”
	Independent term in kernel function	coef0	0.0,0.01,0.1,0.5,1,5,10,50,100
7*Neural network	Hidden layers size	hidden_ayer_ize	(150,100,50,), (100,50,25,), (100,)
	Activation function	activation	tanh’, ’relu’, ’logistic’
	Solver for weight optimization	solver	sgd’, ’adam’
	L2 regularization term on weights	alpha	0.0001,0.001,0.01, 0.05, 0.1, 0.5
	Learning rate	learning_ate	constant’,’adaptive’
	Exponential decay rate for estimates of first moment vector in adam	beta_	0, 0.001, 0.01, 0.1, 0.3, 0.5, 0.9
	Exponential decay rate for estimates of second moment vector in adam	beta_	0, 0.001, 0.01, 0.1, 0.3, 0.5, 0.9
